# Genetic Interactions with Age, Sex, Body Mass Index, and Hypertension in Relation to Atrial Fibrillation: The AFGen Consortium

**DOI:** 10.1038/s41598-017-09396-7

**Published:** 2017-09-12

**Authors:** Lu-Chen Weng, Kathryn L. Lunetta, Martina Müller-Nurasyid, Albert Vernon Smith, Sébastien Thériault, Peter E. Weeke, John Barnard, Joshua C. Bis, Leo-Pekka Lyytikäinen, Marcus E. Kleber, Andreas Martinsson, Henry J. Lin, Michiel Rienstra, Stella Trompet, Bouwe P. Krijthe, Marcus Dörr, Derek Klarin, Daniel I. Chasman, Moritz F. Sinner, Melanie Waldenberger, Lenore J. Launer, Tamara B. Harris, Elsayed Z. Soliman, Alvaro Alonso, Guillaume Paré, Pedro L. Teixeira, Joshua C. Denny, M. Benjamin Shoemaker, David R. Van Wagoner, Jonathan D. Smith, Bruce M. Psaty, Nona Sotoodehnia, Kent D. Taylor, Mika Kähönen, Kjell Nikus, Graciela E. Delgado, Olle Melander, Gunnar Engström, Jie Yao, Xiuqing Guo, Ingrid E. Christophersen, Patrick T. Ellinor, Bastiaan Geelhoed, Niek Verweij, Peter Macfarlane, Ian Ford, Jan Heeringa, Oscar H. Franco, André G. Uitterlinden, Uwe Völker, Alexander Teumer, Lynda M. Rose, Stefan Kääb, Vilmundur Gudnason, Dan E. Arking, David Conen, Dan M. Roden, Mina K. Chung, Susan R. Heckbert, Emelia J. Benjamin, Terho Lehtimäki, Winfried März, J. Gustav Smith, Jerome I. Rotter, Pim van der Harst, J. Wouter Jukema, Bruno H. Stricker, Stephan B. Felix, Christine M. Albert, Steven A. Lubitz

**Affiliations:** 1 Cardiovascular Research Center, Massachusetts General Hospital, Boston, MA USA; 2 Program in Medical and Population Genetics, The Broad Institute of MIT and Harvard, Cambridge, MA USA; 3 National Heart Lung and Blood Institute’s and Boston University’s Framingham Heart Study, Framingham, MA USA; 4 Department of Biostatistics, Boston University School of Public Health, Boston, MA USA; 5 DZHK (German Centre for Cardiovascular Research), partner site: Munich Heart Alliance, Munich, Germany; 6 Institute of Genetic Epidemiology, Helmholtz Zentrum München - German Research Center for Environmental Health, Neuherberg, Germany; 7 Department of Medicine I, University Hospital Munich, Ludwig-Maximilians-University, Munich, Germany; 8 Icelandic Heart Association, 201 Kopavogur, Iceland; 9 Faculty of Medicine, University of Iceland, 101 Reykjavik, Iceland; 10 Population Health Research Institute, Hamilton, Canada; 11 Department of Pathology and Molecular Medicine, McMaster University, Hamilton, Canada; 12 Department of Medicine, Vanderbilt University, Nashville, TN USA; 13 Department of Cardiology, The Heart Centre, Rigshospitalet, Copenhagen, Denmark; 14 Department of Quantitative Health Sciences, Lerner Research Institute, Cleveland Clinic, Cleveland, OH USA; 15 Cardiovascular Health Research Unit, Department of Medicine, University of Washington, Seattle, WA USA; 16 Department of Clinical Chemistry, Fimlab Laboratories and Faculty of Medicine and Life Sciences, University of Tampere, Tampere, Finland; 17 Vth Department of Medicine (Nephrology, Hypertensiology, Endocrinology, Diabetology, Rheumatology), Medical Faculty of Mannheim, University of Heidelberg, Mannheim, Germany; 18 Department of Cardiology, Lund University and Skåne University Hospital, Lund, Sweden; 19 Institute for Translational Genomics and Population Sciences and Department of Pediatrics, Los Angeles Biomedical Research Institute at Harbor-UCLA Medical Center, Torrance, California USA; 20 Division of Medical Genetics, Department of Pediatrics, Harbor-UCLA Medical Center, Torrance, California USA; 21 Department of Cardiology, University of Groningen, University Medical Center Groningen, Groningen, The Netherlands; 22 Department of Cardiology, Leiden University Medical Center, Leiden, The Netherlands; 23 Department of Gerontology and Geriatrics, Leiden University Medical Center, Leiden, The Netherlands; 24 Department of Epidemiology, Erasmus Medical Center, University Medical Center, Rotterdam, The Netherlands; 25 Department of Internal Medicine B, University Medicine Greifswald, Greifswald, Germany; 26 DZHK (German Centre for Cardiovascular Research), partner site Greifswald, Greifswald, Germany; 27 Center for Human Genetic Research, Cardiovascular Research Center, Massachusetts General Hospital, Harvard Medical School, Boston, MA USA; 28 Department of Surgery, Massachusetts General Hospital, Boston, MA USA; 29 Divisions of Preventive Medicine and Genetics, Brigham and Women’s Hospital, Boston, MA USA; 30 Research unit of Molecular Epidemiology, Helmholtz Zentrum München - German Research Center for Environmental Health, Neuherberg, Germany; 31 Institute of Epidemiology II, Helmholtz Zentrum München - German Research Center for Environmental Health, Neuherberg, Germany; 32 Laboratory of Epidemiology and Population Sciences, National Institute on Aging, Intramural Research Program, National Institutes of Health, Bethesda, Maryland 20892 USA; 33 Epidemiological Cardiology Research Center (EPICARE), Wake Forest School of Medicine, Winston Salem, NC USA; 34 Department of Epidemiology, Rollins School of Public Health, Emory University, Atlanta, GA USA; 35 Department of Biomedical Informatics, Vanderbilt University, Nashville, TN USA; 36 Departments of Medicine and Biomedical Informatics, Vanderbilt University, Nashville, TN USA; 37 Department of Medicine, Vanderbilt University Medical Center, Nashville, TN USA; 38 Department of Molecular Cardiology, Lerner Research Institute, Cleveland Clinic, Cleveland, OH USA; 39 Department of Cellular and Molecular Medicine, Lerner Research Institute, Cleveland Clinic, Cleveland, OH USA; 40 Cardiovascular Health Research Unit, Departments of Medicine, Epidemiology and Health Services, University of Washington, Seattle, WA USA; 41 Kaiser Permanente Washington Health Research Institute, Kaiser Foundation Health Plan of Washington, Seattle, WA USA; 42 Cardiovascular Health Research Unit, Division of Cardiology, Departments of Medicine and Epidemiology, University of Washington, Seattle, WA USA; 43 Division of Genomic Outcomes, Department of Pediatrics, Harbor-UCLA Medical Center, Torrance, California USA; 44 Department of Clinical Physiology, Tampere University Hospital and Faculty of Medicine and Life Sciences, University of Tampere, Tampere, Finland; 45 Department of Cardiology, Heart Center, Tampere University Hospital and Faculty of Medicine and Life Sciences, University of Tampere, Tampere, Finland; 46 Department of Clinical Sciences, Lund University, Malmö, Sweden; 47 Department of Internal Medicine, Skåne University Hospital, Malmö, Sweden; 48 Department of Medical Research, Bærum Hospital, Vestre Viken Hospital Trust, Sandvika, Norway; 49 Institute of Health and Wellbeing, University of Glasgow, Scotland, United Kingdom; 50 Robertson Center for Biostatistics, University of Glasgow, Scotland, United Kingdom; 51 Department of Internal Medicine, Erasmus University Medical Center, Rotterdam, The Netherlands; 52 Interfaculty Institute for Genetics and Functional Genomics, University Medicine Greifswald, Greifswald, Germany; 53 Institute for Community Medicine, University Medicine Greifswald, Greifswald, Germany; 54 Division of Preventive Medicine, Brigham and Women’s Hospital, Boston, MA USA; 55 McKusick-Nathans Institute of Genetic Medicine, Johns Hopkins University School of Medicine, Baltimore, MD USA; 56 Population Health Research Institute, McMaster University, Hamilton, Canada; 57 University Hospital Basel, Basel, Switzerland; 58 Cardiovascular Research Institute Basel, Basel, Switzerland; 59 Departments of Medicine, Clinical Pharmacology, and Biomedical Informatics, Vanderbilt University, Nashville, TN USA; 60 Heart and Vascular Institute, Cleveland Clinic, Cleveland, OH USA; 61 Cardiovascular Health Research Unit and Department of Epidemiology, University of Washington, Seattle, WA USA; 62 Clinical Institute of Medical and Chemical Laboratory Diagnostics, Medical University Graz, Graz, Austria; 63 Division of Genomic Outcomes, Departments of Pediatrics and Medicine, Harbor-UCLA Medical Center, Torrance, California USA; 64 Einthoven Laboratory for Experimental Vascular Medicine, LUMC, Leiden, The Netherlands; 65 Interuniversity Cardiology Institute of the Netherlands, Utrecht, The Netherlands; 66 Department of Epidemiology and Internal Medicine, Erasmus University Medical Center Rotterdam, Utrecht, The Netherlands; 67 Inspectorate of Health Care, Utrecht, The Netherlands; 68 Divisions of Preventive Medicine and Cardiovascular Medicine, Brigham and Women’s Hospital, Boston, MA USA

## Abstract

It is unclear whether genetic markers interact with risk factors to influence atrial fibrillation (AF) risk. We performed genome-wide interaction analyses between genetic variants and age, sex, hypertension, and body mass index in the AFGen Consortium. Study-specific results were combined using meta-analysis (88,383 individuals of European descent, including 7,292 with AF). Variants with nominal interaction associations in the discovery analysis were tested for association in four independent studies (131,441 individuals, including 5,722 with AF). In the discovery analysis, the AF risk associated with the minor rs6817105 allele (at the *PITX2* locus) was greater among subjects ≤ 65 years of age than among those > 65 years (interaction p-value = 4.0 × 10^−5^). The interaction p-value exceeded genome-wide significance in combined discovery and replication analyses (interaction p-value = 1.7 × 10^−8^). We observed one genome-wide significant interaction with body mass index and several suggestive interactions with age, sex, and body mass index in the discovery analysis. However, none was replicated in the independent sample. Our findings suggest that the pathogenesis of AF may differ according to age in individuals of European descent, but we did not observe evidence of statistically significant genetic interactions with sex, body mass index, or hypertension on AF risk.

## Introduction

Atrial fibrillation (AF) is a common arrhythmia and is associated with increased risk for stroke, heart failure, and mortality^1–4^. Previous studies have demonstrated that increasing age, male sex, high blood pressure, and obesity are associated with higher AF risk^5–11^. AF is heritable^12–17^, and genetic association studies have identified 16 loci tagged by common genetic variants that are associated with AF^18–22^.

Typically, genome-wide association studies have assumed that the effect of each tested SNP on AF risk is constant across various risk factors, though some data suggest that the effect sizes may differ for different values of risk factors. For example, variants at the *HIATL1* region have been shown to interact with alcohol consumption to affect colorectal cancer risk^23^. Understanding the differences in magnitudes of effect for SNPs in relation to AF across common clinical risk factors could potentially refine our knowledge about the genetic basis of AF in important clinical subsets of individuals. Nevertheless, no large systematic examination of interactions between genetic variants and clinical AF risk factors has been conducted.

We therefore aimed to determine whether common genetic variants interact with age, sex, hypertension, and body mass index to modify AF risk in a large sample of individuals of European ancestry.

## Results

**Figure 1:**
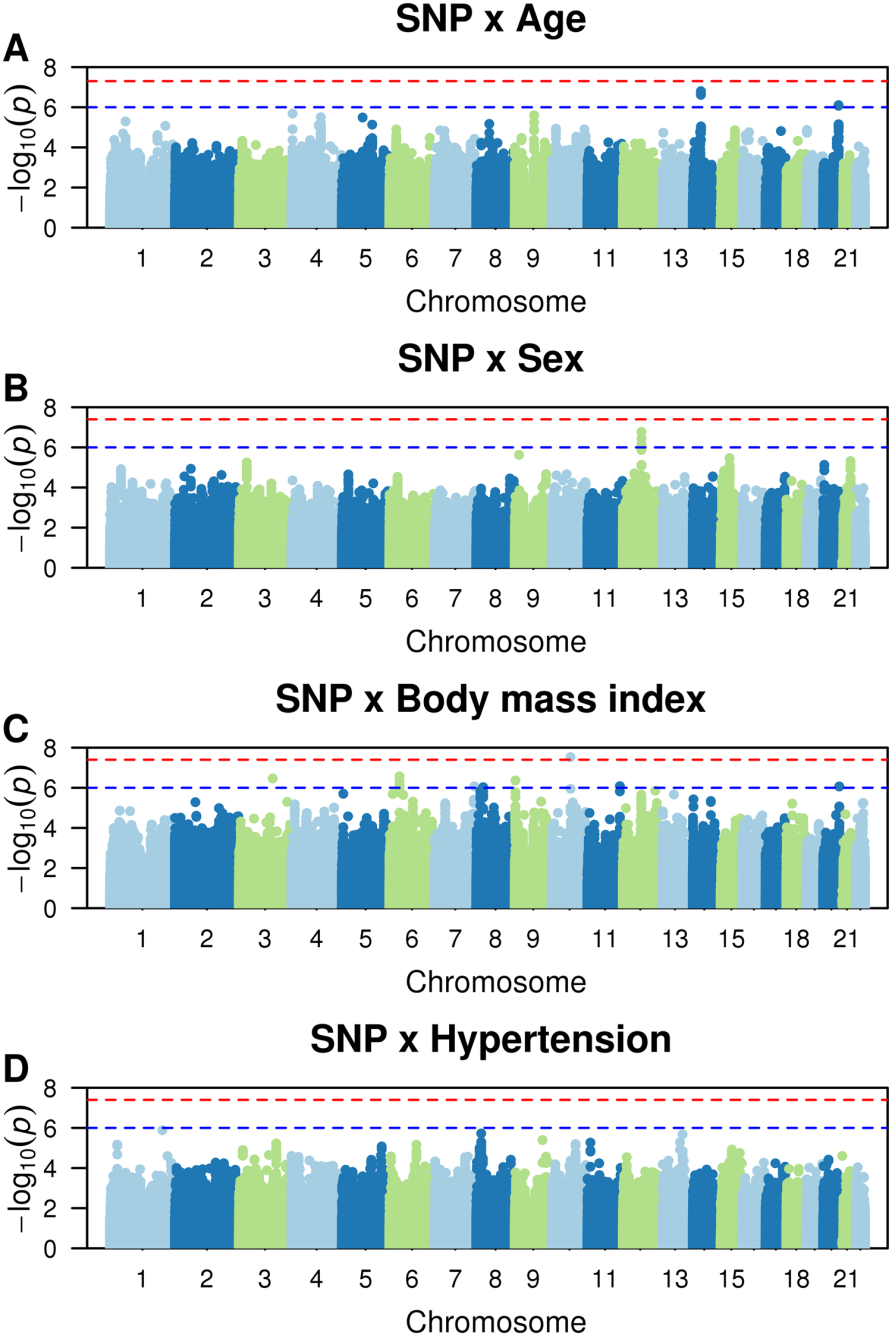
Manhattan plots of genetic interactions with age, sex, body mass index, and hypertension in relation to AF risk. The red line shows the significant interaction p-value threshold (p < 4 × 10^−8^), and the blue line shows the suggestive significant interaction p-value threshold (p < 1 × 10^−6^).

A total of 88,378 subjects, including 7,292 with AF, were included in the discovery analysis (Table 1). The numbers of included SNPs and values of genomic inflation factors (λ) for each study (after applying quality control criteria for SNP exclusions) are displayed in Supplemental Table 1. Overall, genomic inflation factors ranged from 0.85 to 1.2 across studies and interaction analyses. Quantile-quantile (QQ) plots of expected versus observed interaction p-value distributions for associations of the approximately 2.5 million autosomal SNPs for each interaction analysis are displayed in Supplemental Fig. 1a–d. Manhattan plots of -log_10_ (p-value) against the physical coordinates of the 22 autosomes are shown in Fig. 1A–D.

**Table 1:** Subject Characteristics.

	N with AF	N total	Males, n (%)	Age, mean ± SD	Hypertension, n (%)	Body mass index, kg/m^2^, mean ± SD
*Discovery studies*						
Incident AF						
AGES*	158	2718	1011(37.2)	76.3 ± 5.46	2144 (78.9)	76.27 ± 5.46
ARIC*	799	9053	4255 (47.0)	54.3 ± 5.7	2426 (26.8)	27.0 ± 4.8
CHS*	763	3185	1234 (38.7)	72.2 ± 5.3	1680 (52.8)	26.3 ± 4.4
FHS*	306	4025	1751 (43.5)	64.7 ± 12.6	1988 (49.5)	27.7 ± 5.2
MESA*	155	2526	1206 (47.74)	62.66 ± 10.24	975 (38.6)	27.74 ± 5.06
PREVEND*	113	3520	1811 (50)	49.5 ± 12.4	1157 (30)	26.1 ± 4.3
PROSPER*	505	5244	2524 (48.1)	75.34 ± 3.35	3257 (62.1)	26.82 ± 4.18
RS*	591	5665	2282 (40.3)	69.1 ± 8.98	3081 (54.4)	26.32 ± 3.69
WGHS*	648	20842	0 (0)	54.6 ± 7.0	5022 (24)	25.3 ± 6.7
Prevalent AF						
AFNET/KORA	448	886	524 (59.1)	53.4 ± 7.8	326 (36.8)	27.9 ± 4.6
AGES	241	2959	1154 (39.0)	76.47 ± 5.50	2359 (79.8)	27.06 ± 4.44
BioVU o1	238	4766	2552 (53.6)	62.2 ± 16.3	3270 (68.6)	26.2 ± 11.2
BioVU 660	120	3790	1722 (45.4)	62.8 ± 15.9	1966 (51.9)	24.0 ± 15.0
CCAF	807	2661	1918 (72.1)	61.7 ± 11.15	1793 (67.4)	29.5 ± 5.78
FHS	253	4401	1957 (44.5)	65.4 ± 12.8	2215 (50.5)	27.70 ± 5.16
LURIC	361	2959	2077 (70.2)	63.0 ± 10.6	2154 (72.8)	27.5 ± 4.02
MGH/MIGEN	366	1277	780 (61.1)	49.5 ± 9.7	—	—
RS	309	5974	2427 (40.6)	69.4 ± 9.1	3273 (54.8)	26.3 ± 3.69
SHIP	107	1923	927 (48.2)	50.97 ± 15.07	496 (25.8)	27.33 ± 4.56
*Replication studies*						
Incident AF						
MDCS	876	7353	3800 (48)	58.8 ± 6.6	5010 (68)	26.1 ± 4.1
Prevalent AF						
BEAT-AF	1520	3040	1795 (59)	51.7 ± 18.6	1363 (45)	25.8 ± 4.4
FINCAVAS	940	3021	1835 (61)	61.9 ± 14	2117 (70)	27.5 ± 4.5
UK Biobank	2386	118027	55669 (47)	56.9 ± 7.9	25307 (21)	27.5 ± 4.8

Abbreviations: AF: atrial fibrillation; NA: not available; SD: standard deviation. *Information at DNA collection.

### Interactions with risk factors at known AF loci

We first evaluated the associations between genetic interactions and clinical factors (age, sex, hypertension, and body mass index) with AF at 16 established AF susceptibility loci from prior genome-wide association studies (Supplemental Table 2; significance threshold = 6.25 × 10^−4^, see methods for explanation). We observed significant interactions with age for SNP rs6817105 (upstream of *PITX2* at chromosome locus 4q25; interaction p-value = 4 × 10^−5^; Table 2). The minor C allele of SNP rs6817105 was associated with a greater risk for AF among individuals 65 years of age or younger [odds ratio (OR) = 1.75, 95% CI 1.61–1.91, p = 6.2 × 10^−36^], than among participants older than 65 years (OR = 1.38, 95% CI 1.28–1.47, p = 6.3 × 10^−17^). Among other known AF loci, SNP rs3807989 at the *CAV1* locus displayed a nominal interaction with age that was not statistically significant (interaction p = 2.9 × 10^−3^; Table 2). However, the major G allele was associated with higher AF risk in the younger group (OR = 1.25, 95% CI 1.16–1.34, p = 3.6 × 10^−10^ for subjects ≤ 65 years; OR = 1.09, 95% CI 1.03–1.15, p = 1.4 × 10^−3^ for subjects >65). We did not observe any significant interactions between AF-associated SNPs and sex, hypertension, or body mass index.

**Table 2:** Multiplicative SNP interactions with AF risk factors at known AF loci.

SNP	A1/A2	A1 freq	Loc	Closest gene	SNP and AF risk factor interaction							
					Age		Sex		Body mass index		Hypertension	
					Interaction β *(se)	p	Interaction β (se)	p	Interaction β (se)	p	Interaction β (se)	p
rs6666258	C/G	0.30	1q21	*KCNN3*	0.0979 (0.048)	0.04	0.0092 (0.045)	0.84	−0.0023 (0.005)	0.62	0.024 (0.045)	0.60
rs3903239	G/A	0.44	1q24	*PRRX1*	0.0661 (0.044)	0.13	−0.050 (0.041)	0.23	0.0021 (0.004)	0.63	−0.014 (0.041)	0.74
rs4642101	G/T	0.65	3p25	*CAND2*	0.0828 (0.047)	0.08	0.0425 (0.045)	0.35	−0.0024 (0.005)	0.59	0.0813 (0.045)	0.07
rs1448818	C/A	0.25	4q25	*PITX2*	0.0207 (0.049)	0.67	0.0285 (0.046)	0.54	0.0094 (0.005)	0.04	−0.0597 (0.045)	0.19
rs6817105	C/T	0.13	4q25	*PITX2*	0.2420 (0.059)	4.0 × 10^−5^	0.0065 (0.055)	0.91	0.0078 (0.006)	0.16	−0.0516 (0.055)	0.35
rs4400058	A/G	0.09	4q25	*PITX2*	0.0665 (0.070)	0.34	−0.0343 (0.068)	0.61	−0.0051 (0.007)	0.47	−0.0406 (0.066)	0.54
rs6838973	C/T	0.57	4q25	*PITX2*	0.0636 (0.045)	0.16	−0.0599 (0.043)	0.16	−0.0005 (0.004)	0.90	−0.0823 (0.042)	0.05
rs13216675	T/C	0.69	6q22	*GJA1*	−0.0287 (0.050)	0.57	0.0869 (0.047)	0.07	0.0064 (0.005)	0.18	0.0616 (0.046)	0.18
rs3807989	G/A	0.60	7q31	*CAV1*	0.1329 (0.045)	2.9 × 10^−3^	−0.0054 (0.041)	0.90	−0.0003 (0.004)	0.95	−0.0603 (0.041)	0.14
rs10821415	A/C	0.42	9q22	*C9orf3*	0.0736 (0.047)	0.11	−0.0039 (0.044)	0.93	0.0012 (0.004)	0.79	−0.0813 (0.043)	0.06
rs10824026	A/G	0.84	10q22	*SYNPO2L*	−0.0035 (0.063)	0.96	−0.0213 (0.059)	0.72	0.0139 (0.006)	0.02	0.258 (0.060)	0.66
rs12415501	T/C	0.16	10q24	*NEURL*	0.0701 (0.064)	0.27	0.0994 (0.058)	0.09	0.0011 (0.006)	0.85	0.0684 (0.057)	0.23
rs10507248	T/G	0.73	12q24	*TBX5*	−0.0573 (0.050)	0.25	0.0571 (0.046)	0.22	−0.0025 (0.005)	0.59	−0.0208 (0.046)	0.65
rs1152591	A/G	0.48	14q23	*SYNE2*	0.0178 (0.045)	0.69	−0.0082 (0.042)	0.85	0.004 (0.004)	0.35	0.0255 (0.042)	0.54
rs7164883	G/A	0.16	15q24	*HCN4*	−0.0294 (0.058)	0.61	0.0284 (0.054)	0.60	0.0016 (0.006)	0.78	−0.0271 (0.055)	0.62
rs2106261	T/C	0.18	16q22	*ZFHX3*	0.0106 (0.057)	0.85	0.0434 (0.054)	0.42	−0.0021 (0.006)	0.71	0.110 (0.053)	0.04

The significance threshold 0.01/16 = 6.25 × 10^−4^. Abbreviations: AF: atrial fibrillation; A1: allele 1; the risk allele was defined based on a prior GWAS^56^; A2: allele 2; A1 freq: allele 1 frequency; Loc: locus; p: P-value for the interaction between the risk factor and the SNP.

*Interaction β was from regression using an additive model. Interaction β (se) was calculated as the meta-analysis log(effect) in subjects ≤ 65 years of age minus the meta-analysis log(effect) in subjects >65 years of age, or as the multiplicative interaction between SNP*risk factor for sex (females vs. males), hypertension (hypertensive vs. not), and body mass index (per 1 unit increment).

### Interactions with risk factors in genome-wide analyses

Table 3 displays the results for SNP interactions with AF risk factors across the genome. The most significant genetic interaction that exceeded our genome-wide significance threshold (an interaction p-value < 4 × 10^−8^, see methods for explanation) was observed for SNP rs12416673 with body mass index (interaction p = 2.9 × 10^−8^; 6.4 kb upstream of *COL13A1* at chromosome region 10q21; Table 3; Supplemental Figure 2). Specifically, with each 1-unit increase in body mass index, each copy of the minor A allele of SNP rs12416673 was associated with an increased risk for AF (interaction β = 0.0224, interaction p = 2.9 × 10^−8^). Additionally, we observed 8 loci that exhibited suggestive interactions with AF risk factors (i.e., the interaction p-value was < 1 × 10^−6^ for the top SNP, and two or more SNPs in the same region exhibited interaction p-values < 1 × 10^−5^). Specifically, we observed interactions with age at 2 loci, sex at 1 locus, and body mass index at 5 loci (Table 3). No genetic interactions with hypertension exceeded the suggestive genome-wide or adjusted AF susceptibility locus significance thresholds.

**Table 3:** Discovery and replication analysis results of top SNP interactions with AF risk factors.

SNP	Loc	Closest gene	A1/A2	Discovery			Replication			Combined		
				A1 freq (%)	Interaction β (se)	p	A1 freq (%)	Interaction β (se)*	p	A1 freq (%)	Interaction β (se)*	p
***SNP x Age***												
rs6817105^†^	4q25	*PITX2*	C/T	0.13	0.2420 (0.059)	4.0 × 10^−5^	0.11	0.2213 (0.067)	9.5 × 10^−4^	0.12	0.2420 (0.043)	1.7 × 10^−8^
rs3807989^†^	7q31	*CAV1*	G/A	0.60	0.1329 (0.045)	2.9 × 10^−3^	0.59	−0.0531 (0.050)	0.28	0.59	0.0325 (0.032)	3.1 × 10^−1^
rs2356251	14q22	*MAP4K5*	C/G	0.06	0.5716 (0.109)	1.6 × 10^−7^	0.04	−0.1894 (0.123)	0.12	0.05	0.2294 (0.081)	4.5 × 10^−3^
rs1572779	20q13	*MIR548AG2*	G/T	0.10	0.3468 (0.070)	7.9 × 10^−7^	0.09	0.1456 (0.090)	0.10	0.10	0.2446 (0.054)	5.7 × 10^−6^
***SNP x Sex***												
rs2730668	12q21	*TRHDE*	T/C	0.76	0.2734 (0.052)	1.7 × 10^−7^	0.76	0.0846 (0.0563)	0.13	0.76	0.1860 (0.0383)	1.2 × 10^−6^
***SNP x Body Mass Index***												
rs9394492	6q21	*BTBD9*	T/C	0.36	0.0222 (0.004)	2.7 × 10^−7^	0.38	−0.0070 (0.005)	0.15	0.37	0.0092 (0.003)	4.1 × 10^−3^
rs1874425	8q21	*ADrA1A*	T/C	0.25	0.0231 (0.005)	9.3 × 10^−7^	0.25	0.0070 (0.005)	0.18	0.25	0.0160 (0.004)	5.4 × 10^−6^
rs1545567	9p24	*VLDLR*	T/C	0.64	−0.0256 (0.005)	4.3 × 10^−7^	0.65	−0.0010 (0.005)	0.85	0.65	−0.0131 (0.004)	2.3 × 10^−4^
rs12416673	10q21	*COL13A1*	A/G	0.43	0.0224 (0.004)	2.9 × 10^−8^	0.43	−0.0018 (0.005)	0.71	0.43	0.0122 (0.003)	7.1 × 10^−5^
rs6062828	20q13	*LOC105372719/YTHDF1*	C/G	0.68	−0.0245 (0.005)	8.6 × 10^−7^	0.69	−0.0105 (0.005)	0.03	0.69	−0.0174 (0.004)	6.5 × 10^−7^

Abbreviations: AF: atrial fibrillation; A1: allele 1; the risk allele was defined based on a prior GWAS^56^; A2: allele 2; A1 freq: allele 1 frequency; Loc: locus; p: P-value for the interaction between the risk factor and the SNP.

*Interaction β was from regression using additive model. Interaction β (se) was calculated as the meta-analysis log(effect) in subjects ≤ 65 years of age minus the meta-analysis log(effect) in subjects > 65 years of age, or as the multiplicative interaction between SNP* risk factor for sex (females vs. males) and body mass index (per 1 unit increment).

^†^Known AF loci.

### Replication

**Figure 2:**
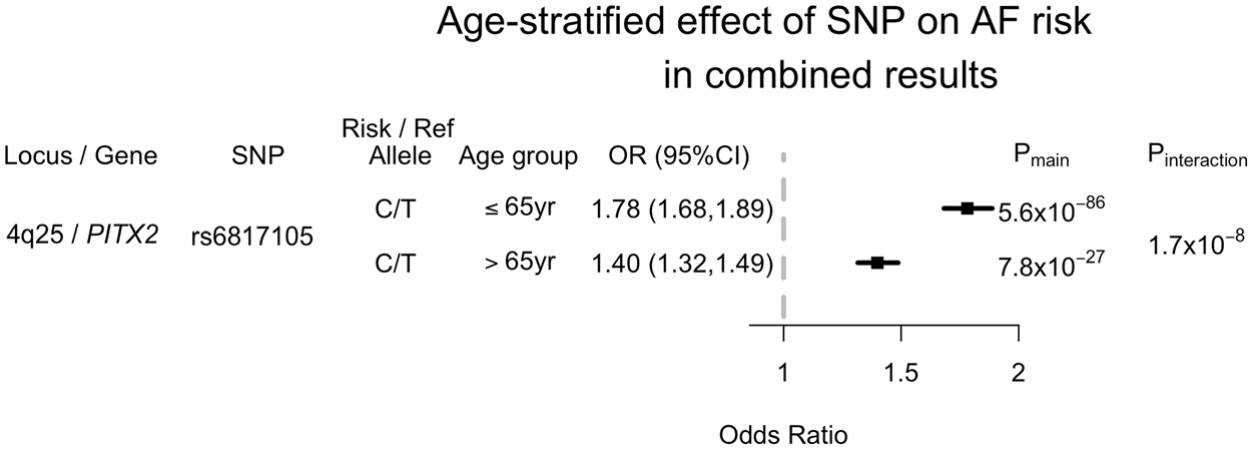
Age-stratified association between the chromosome 4q25 locus and AF in the combined dataset of primary and replication studies. OR and P_main_ refer to the odds ratio and p-value for the association test between rs6817105 and AF risk in each age-stratum. P_interaction_ refers to the p-value corresponding to the difference in effect sizes between the two age strata tested.

In total, we selected 10 SNP interactions (Table 3) for replication association testing in four independent cohorts (131,441 individuals, including 5,722 with AF). Only one interaction remained significantly associated with AF. SNP rs6817105 at the 4q25 locus exhibited a significant interaction with age (interaction p = 9.5 × 10^−4^). As in our discovery analysis, among individuals with the minor C allele of rs6817105, those ≤65 years old had a greater risk for AF (OR = 1.80; 95% CI 1.67–1.95, p = 6.6 × 10^−52^), than participants older than 65 years (OR = 1.45; 95% CI 1.30–1.61, p = 1.4 × 10^−11^). Similarly, rs6817105 was associated with a 27% higher AF risk in subjects ≤65 years of age (compared with subjects >65 years of age) in the combined discovery and replication analysis (interaction p = 1.7 × 10^−8^; Fig. 2). A greater risk of AF for the rs6817105 C allele was observed in participants aged 65 years or younger (OR = 1.78; 95% CI 1.68–1.89, p = 5.6 × 10^−86^) than in participants older than 65 years (OR = 1.40; 95% CI 1.32–1.49, p = 7.8 × 10^−27^).

### Power calculation

**Figure 3:**
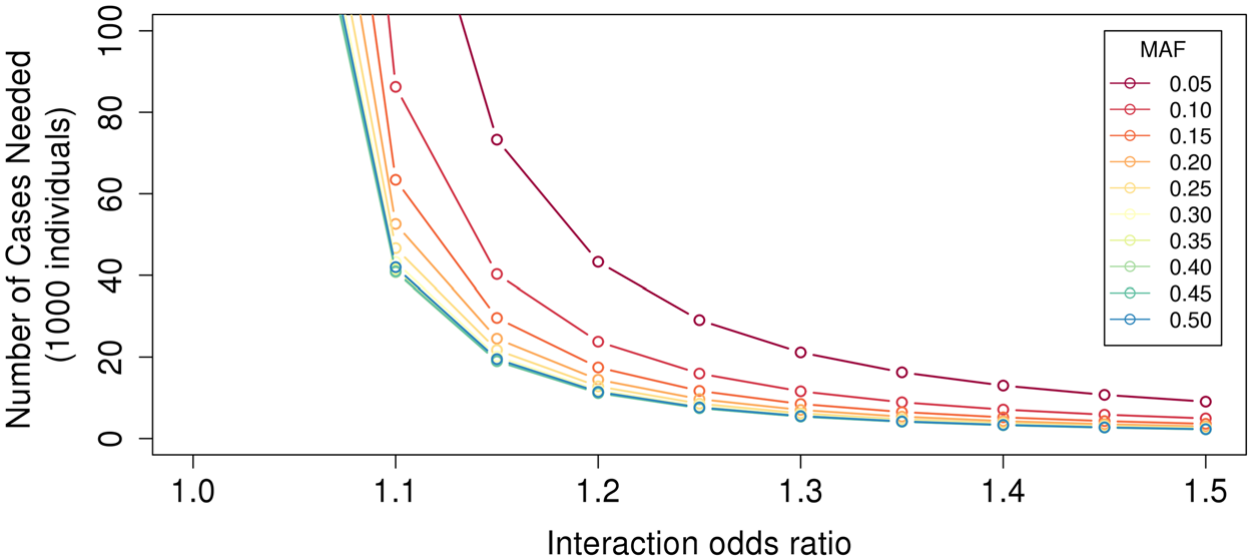
Number of cases required to detect interaction odds ratios between 1.01 to 1.5 with common SNPs (minor allele frequencies (MAF) of 0.05–0.5) with 80% power assuming an AF prevalence of 1%, 50% males, SNP marginal effect odds ratios of 1.5, sex marginal effect odds ratios of 1.5, case:control ratios of 1:10, and α = 4 × 10^−8^. Power calculations were performed using Quanto^24^.

Given the lack of observed associations between SNP interactions with clinical risk factors and AF, we performed power calculations to estimate power for discovery using Quanto^24^ (http://biostats.usc.edu/Quanto.html; Fig. 3). As an example, we estimated power to observe a SNP interaction with sex, assuming a population comprised of 50% males, an AF population prevalence of 1%, and a case to control ratio of 1:10 (as in our study). We modeled a main effect OR of 1.5 for sex, and a genetic odds ratio of 1.5 for a SNP. We estimated that >100,000 AF cases would be a required to achieve 80% power for such an effect size, indicating that we had limited power to detect all but substantial genetic interactions with clinical risk factors.

## Discussion

In our analysis of ~88,000 individuals of European ancestry, including 7,292 individuals with AF, we observed that the well-established AF locus at chromosome region 4q25 (tagged by rs6817105) was associated with a differential risk for AF according to age. Specifically, the OR for each copy of the minor rs6817105 allele was 1.78 for individuals ≤65 years of age, compared to 1.40 for individuals >65 years. Beyond the age interaction with the 4q25 locus, we did not observe any significant interactions between genetic variants and age, sex, hypertension, or body mass index after replication attempts. These findings suggest that strong genetic interactions with the AF risk factors studied in this manuscript are unlikely to be prominent mechanisms driving AF susceptibility.

Our findings support and extend prior studies examining genetic interactions for AF. For example, top variants at the 4q25 chromosome locus, upstream of *PITX2*, were associated with greater AF risks among younger individuals in secondary analyses of a genome-wide association study^20^. However, no formal statistical test of interaction was performed. Greater effect sizes of other AF susceptibility SNPs at the 4q25 locus were also observed among younger rather than older individuals in some, but not all, cohorts in a large replication study^25^. Moreover, in keeping with our observations, prior studies did not find evidence that AF risk is modified by interactions between SNPs at the 4q25 locus and sex^20^. Our findings demonstrate that genetic variation at the 4q25 locus is, on average, associated with greater risks for early-onset AF.

Our findings are consistent with epidemiologic observations demonstrating greater heritability for earlier onset of AF^16^. The stronger effect of the 4q25 locus on AF in a younger population implies that the contribution of this locus to AF susceptibility may be more relevant to those with early-onset AF, rather than later onset forms. Overall, our observation that genetic variation at the 4q25 locus is associated with AF (beyond genome-wide significant thresholds) in both younger and older individuals underscores the predominant role of this locus in AF pathogenesis–regardless of age.

*PITX2* is a homeobox transcription factor involved in specification of pulmonary vasculature^26^, cardiac laterality^27^, and suppression of a left atrial sinoatrial-node like pacemaker^28^. Heterozygous null *Pitx2c* mouse hearts are more susceptible to pacing induced AF than are wild-type counterparts^29^. The relative roles of *PITX2* regulation in AF susceptibility in both human development and in adult life are unclear. Future larger studies are warranted to systematically determine whether there are different age-specific etiologic subtypes of AF, and whether *PITX2* modulation varies with age according to genotype.

Although our analysis was not designed to specifically quantify the contribution of genetic factors to AF heritability, the absence of observed interactions between AF and sex, body mass index, and hypertension suggests that common variant interactions with these clinical risk factors are unlikely to explain a substantial proportion of variance in AF susceptibility. Larger studies will be necessary to accurately quantify the contributions of both common and rare variation, epigenetic mechanisms, copy-number variation, epistatic effects, and other environmental interactions that may influence AF heritability. Moreover, further examination is needed to determine the extent to which the 4q25 locus, the predominant susceptibility locus for AF, explains the heritability of the condition.

Our study should be interpreted in the context of the study design. First, we included individuals of European ancestry only, so our finding may not be generalizable to other racial groups. Second, AF risk factors were available only at the time of AF onset in case-control studies, rather than before AF onset, potentially biasing toward the null any biologically relevant SNP by risk factor interactions that may occur years before the onset of AF. However, we suspect that such misclassification of risk factor status is unlikely to have resulted in systematic bias for body mass index (which tends to be relatively stable over time^30^) and age (because an interaction with age and the *PITX2* locus is supported by prior observations). Third, our sample size provided limited power to identify interactions with relatively small effect sizes. Additionally, the use of more powerful statistical approaches^31^, non-multiplicative interactions, and inclusion of additional AF risk factors, may facilitate identification of loci at which genetic interactions exist in relation to AF. Fourth, our single SNP interaction approach does not exclude a lack of interaction with polygenic susceptibility to AF. Fifth, we acknowledge that AF may be clinically unrecognized, leading to misclassification of AF status, and that we lacked power to analyze AF subtypes separately. Future analyses with additional arrhythmia outcomes may help clarify the role of genetic interactions with risk factors across a range of arrhythmia phenotypes.

In summary, we identified a significant interaction with age at the AF susceptibility locus on chromosome 4q25 upstream of *PITX2* in individuals of European ancestry. Despite several suggestive SNP interactions with common AF risk factors in discovery analyses, we did not observe substantial evidence for such interactions as common mechanisms underlying AF risk.

## Methods

### Study population

Discovery cohorts included the: German Competence Network for Atrial Fibrillation and Cooperative Health Research in the Region Augsburg (**AFNET/KORA**); Age, Gene/Environment Susceptibility Reykjavik Study (**AGES**) study; Atherosclerosis Risk in Communities (**ARIC**) study; Vanderbilt electronic medical record-linked DNA repository (**BioVU**); Cleveland Clinic Lone AF study (**CCAF**); Cardiovascular Health Study (**CHS**); Framingham Heart Study (**FHS**); Ludwigshafen Risk and Cardiovascular Health (**LURIC**) study; Multi-Ethnic Study of Atherosclerosis **(MESA)**; Massachusetts General Hospital Lone AF study and Myocardial Infarction Genetics Consortium (**MGH/MIGEN**); Prevention of Renal and Vascular End-stage Disease (**PREVEND**) study; PROspective Study of Pravastatin in the Elderly at Risk (**PROSPER**); Rotterdam Study (**RS**); Study of Health in Pomerania (**SHIP**); and Women’s Genome Health Study (**WGHS**). Replication studies included the: Basel Atrial Fibrillation Cohort Study (**Beat-AF**); Finnish Cardiovascular Study (**FINCAVAS**); Malmo diet and cancer study (**MDCS**); and **UK Biobank**. Detailed descriptions of each study have been previously reported (Supplemental Methods and Supplemental Table 3).

The study protocol was approved by the Ethical Committee/institutional review boards of Ludwig Maximilian University of Munich, National Bioethics Committee, Johns Hopkins Bloomberg School of Public Health, University of Minnesota, Vanderbilt University Medical Center, Cleveland Clinic, University of Washington, Boston University Medical Campus, Rhineland-Palatinate State Chamber of Physicians, Massachusetts General Hospital, University Medical Center Groningen, Leiden University Medical Center, Erasmus MC - University Medical Center Rotterdam, University Medicine Greifswald, Brigham and Women’s Hospital, ethics committee northwest/central Switzerland, ethics committee Zurich, Pirkanmaa Hospital District, and Lund University. All MESA study sites received approval to conduct this research from local institutional review boards at: Columbia University (for the MESA New York Field Center), Johns Hopkins University (for the MESA Baltimore Field Center), Northwestern University (for the MESA Chicago Field Center), University of California, Los Angeles (for the MESA Los Angeles Field Center), University of Minnesota (for the MESA Twin Cities Field Center), Wake Forest University Health Sciences Center (for the MESA Winston-Salem Field Center). Written informed consent was obtained from all study subjects or their proxies (except BioVU, which is a de-identified EMR biorepository and was “opt-out” prior to December 2014). All experiments were performed in accordance with relevant guidelines and regulations.

### AF ascertainment

Ascertainment of AF and risk factors in each study has been described previously^10, 14, 32–54^, Detailed descriptions are provided in Supplemental Table 3. We defined prevalent AF as an event that was diagnosed at or prior to an individual’s DNA collection in cohort studies and on the basis of AF ascertainment in case-control studies. We defined incident AF as an event that was diagnosed after DNA collection among participants free of clinically apparent AF at DNA collection in cohort studies. All AF risk factors except age were ascertained at the time of DNA collection. Age was defined at DNA collection or at the date of recruitment in cohort studies, and at time of AF diagnosis (for AF cases) or at time of DNA collection (for controls) in case-control studies.

### Exposure ascertainment

Sex was defined on the basis of self-report. Participants were classified as having hypertension if the systolic blood pressure was ≥140 mm Hg or the diastolic blood pressure was ≥90 mm Hg at any clinic visit or exam antecedent to DNA collection, or if the participant was receiving treatment with an antihypertensive medication and had a self-reported history of hypertension or high blood pressure at the time of DNA collection (not applicable in ARIC or FHS; Supplemental Table 3). Body mass index was defined as the weight (kg) divided by the height (m) squared. Blood pressure measurements, medication lists, weights, and heights were ascertained according to study-specific protocols. All participants in the discovery analysis were genotyped on genome-wide SNP array platforms (Supplemental Table 4). Imputed genotypes used in our analysis included approximately 2.5 million genetic variants from the HapMap CEU sample (release 22).

### Statistical analysis

For each individual study, logistic regression (for prevalent AF; for incident AF in **MESA** and **PREVEND** only), generalized estimating equations (in **FHS** to account for related individuals), or Cox proportional hazard regression (for incident AF in prospective cohorts other than **MESA** and **PREVEND**) were performed to examine whether AF was associated with interactions between SNP and AF risk factors. For Cox models, person-time began at study baseline, and individuals were censored at death or loss to follow-up. Robust variance estimates were used when feasible. Details of the regression models are described in Supplemental Table 4. All models were adjusted for age (age at baseline for incident AF, and age at AF onset for prevalent AF), sex, site (ARIC and CHS), sub-cohort (FHS), study-specific covariates, and population structure, if applicable. SNPs with low imputation quality (R-square < 0.3) or a minor allele frequency < 0.05 were removed from the analysis.

For interaction analyses involving sex, hypertension, and body mass index, main effect terms for each risk factor, as well as multiplicative interaction terms between each SNP and the respective risk factor, were included in the regression models. Regarding analyses of age, nonlinear associations between SNPs and age could potentially go undetected, due to variable distributions of age across the studies in our analysis. Additionally, some studies had only or mostly early-onset/late-onset AF cases, which limited our ability to perform a regression model with dichotomized age in such samples. Therefore, we assessed SNP interactions with age by comparing meta-analysis estimates of associations between each SNP and AF in individuals ≤65 versus >65 years of age (see below). Studies with <100 AF events in each stratum of age were not included, in order to avoid unstable effect estimates.

Estimators for multiplicative interaction terms were meta-analyzed for sex, hypertension, and body mass index analyses in METAL^55^, using an inverse-variance weighted fixed-effects approach with genomic-control correction. For age, we performed an inverse-variance weighted fixed-effects meta-analysis of the estimators for each SNP separately within each age stratum, with genomic-control correction. Estimators were compared using a Z test, as mentioned above.

SNPs with absolute effect sizes ≥3 or SNPs that were available in only one study were excluded from our final results, to minimize the likelihood of spurious false positive findings. For each of the four genome-wide interaction assessments, we employed an experiment-wide two sided alpha threshold of 0.05, which we adjusted for multiple hypothesis testing. We distributed the alpha differentially across the genome, according to *a priori* hypotheses about interactions between SNPs and AF risk factors. Specifically, we distributed one-fifth of the alpha to each of the 16 most significantly associated SNPs at genome-wide significant loci identified in prior studies^56, 57^ (interaction p < 0.01/16 = 6.25 × 10^−4^). The remaining four-fifths of the alpha were distributed evenly across the genome, for an alpha threshold of 4 × 10^−8^ (interaction p < 0.04/~1,000,000 independent tests).

Significantly associated SNPs and SNPs with suggestive associations (i.e., an interaction p < 0.005 at a recognized AF GWAS locus; or an interaction p < 1 × 10^−6^ combined with interaction p < 1 × 10^−5^ for two additional SNPs within the same ±50 kb region) in the discovery analysis were carried forward for replication testing. In total, we carried forward 10 SNPs for replication testing (see below), and therefore assumed a replication interaction p threshold of 0.005 (0.05/10 SNPs). The results of replication studies alone, as well as combined with results from discovery studies, were meta-analyzed as described above.

## Electronic supplementary material

Supplementary information 
