# Supplementary material for: Genetic Interactions with Age, Sex, Body Mass Index, and Hypertension in Relation to Atrial Fibrillation: The AFGen Consortium
Source: Sci Rep. 2017 Sep 12;7:11303. doi: 10.1038/s41598-017-09396-7 (PMC5595875; doi:10.1038/s41598-017-09396-7)
Supplement: Supplementary file 1 — Supplementary information [file 41598_2017_9396_MOESM1_ESM.doc]

**Genetic Interactions with Age, Sex, Body Mass Index, and Hypertension in Relation to Atrial Fibrillation: The AFGen Consortium**

Lu-Chen Weng, Kathryn L. Lunetta, Martina Müller-Nurasyid, Albert Vernon Smith, Sébastien Thériault, Peter E. Weeke, John Barnard, Joshua C. Bis, Leo-Pekka Lyytikäinen, Marcus E. Kleber, Andreas Martinsson, Henry J. Lin, Michiel Rienstra, Stella Trompet, Bouwe P. Krijthe, Marcus Dörr, Derek Klarin, Daniel I. Chasman, Moritz F. Sinner, Melanie Waldenberger, Lenore J. Launer, Tamara B. Harris, Elsayed Z. Soliman, Alvaro Alonso, Guillaume Paré, Pedro L. Teixeira, Joshua C. Denny, M. Benjamin Shoemaker, David R. Van Wagoner, Jonathan D. Smith, Bruce M Psaty, Nona Sotoodehnia, Kent D. Taylor, Mika Kähönen, Kjell Nikus, Graciela E. Delgado, Olle Melander, Gunnar Engström, Jie Yao, Xiuqing Guo, Ingrid E. Christophersen, Patrick T. Ellinor, Bastiaan Geelhoed, Niek Verweij, Peter Macfarlane, Ian Ford, Jan Heeringa, Oscar H. Franco, André G. Uitterlinden, Uwe Völker, Alexander Teumer, Lynda M. Rose, Stefan Kääb, Vilmundur Gudnason, Dan E. Arking, David Conen, Dan M. Roden, Mina K. Chung, Susan R. Heckbert, Emelia J. Benjamin, Terho Lehtimäki, Winfried März, J. Gustav Smith, Jerome I. Rotter, Pim van der Harst, J. Wouter Jukema, Bruno H. Stricker, Stephan B. Felix, Christine M. Albert, and Steven A. Lubitz

**Index of Supplemental Material**

| **Page** | **Section** |
| --- | --- |
| **3** | Supplemental Methods. |
| **8** | Supplemental Table 1. Analysis-specific numbers of analyzed autosomal SNPs and genomic inflation estimates by study sample. |
| **9** | Supplemental Table 2. Sixteen AF-associated loci reported in previous association studies. |
| **10** | Supplemental Table 3. Details of study population. |
| **12** | Supplemental Table 4. Details regarding study genotyping and data analysis. |
| **14** | Supplemental Figure 1a-1d. Quantile-quantile plots for each genetic interaction analysis. |
| **15** | Supplemental Figure 2. Regional Association Plot for Genome-wide Significant SNPs by BMI interaction on Chromosome 10. |
| **16** | Supplemental References |

**SUPPLEMENTAL METHODS**

***Description of study cohorts***

The following studies included in the current analysis have been described elsewhere: the **Age, Gene/Environment Susceptibility Study (AGES) Reykjavik study**,[^1^](#_ENREF_1) the **Arrhythmia-Biobank-LMU** (**AFLMU**, formerly known as **AFNET**) and the **Cooperative Health Research in the Region of Augsburg (KORA)**,[^1^](#_ENREF_1) the **Atherosclerosis Risk in Communities** **(ARIC) study**,[**^1^**](#_ENREF_1) **Cleveland Clinic Lone Atrial Fibrillation GeneBank Study (CCAF)**,[^1^](#_ENREF_1) the **Cardiovascular Health Study (CHS)**,[^1^](#_ENREF_1) **Framingham Heart Study (FHS)**,[^1^](#_ENREF_1) **Massachusetts General Hospital (MGH) AF study**,[^1^](#_ENREF_1) the **Rotterdam Study (RS)**,[^1^](#_ENREF_1) the **Study of Health in Pomerania (SHIP)**,[^1^](#_ENREF_1) the **Women’s Genome Health Study (WGHS)**,[^1^](#_ENREF_1) **a**nd the **PROspective Study of Pravastatin in the Elderly at Risk (PROSPER)**.[^2^](#_ENREF_2) Additional studies were described here:

***Discovery studies***

**BioVU**

BioVU is the Vanderbilt University Medical Center's biorepository linked to de-identified electronic health records. BioVU operations[^3^](#_ENREF_3) and ethical oversight[^4^](#_ENREF_4) been described elsewhere. Briefly, DNA is collected from discarded blood samples remaining after routine clinical testing at Vanderbilt outpatient clinics in Nashville, Tennessee and surrounding areas, and is linked to a de-identified version of the patient's electronic health record termed the “Synthetic Derivative.” AF cases were defined as individuals who were aged >18 years, had an ICD-9 diagnosis for AF or flutter (ICD-9: 427.3, 427.31, and 427.32), or a cardiologist diagnosis of AF as identified by a natural language processing tool from the unstructured free text of the ECG impression. In all instances, patients with a history of a heart transplant were excluded (Current Procedural Terminology: 33935, 3394, and 580; ICD-9: V42.1, 996.83).[^5^](#_ENREF_5)

**LURIC**

The Ludwigshafen Risk and Cardiovascular Health (LURIC) study is an ongoing prospective study of more than 3,300 individuals of German ancestry in whom cardiovascular and metabolic phenotypes (CAD, MI, dyslipidemia, hypertension, metabolic syndrome and diabetes mellitus) have been defined or ruled out using standardized methodologies in all study participants.^184^ Inclusion criteria for LURIC were: German ancestry (limitation of genetic heterogeneity), clinical stability (except for acute coronary syndromes) and availability of a coronary angiogram. Exclusion criteria were: any acute illness other than acute coronary syndromes, any chronic disease where non-cardiac disease predominated and a history of malignancy within the last five years. Genome-wide analyses using the Affymetrix 6.0 have been completed in all participants. A 10-year clinical follow-up for total and cause specific mortality has been completed.

**MESA**

The Multi-Ethnic Study of Atherosclerosis (MESA) is a study of the characteristics of subclinical cardiovascular disease (disease detected non-invasively before it has produced clinical signs and symptoms) and the risk factors that predict progression to clinically overt cardiovascular disease or progression of the subclinical disease. The cohort is a diverse, population-based sample of 6,814 asymptomatic men and women aged 45-84. Approximately 38 percent of the recruited participants are white, 28 percent African-American, 22 percent Hispanic, and 12 percent Asian (predominantly of Chinese descent). Participants were recruited during 2000-2002 from 6 field centers across the U.S. (at Wake Forest University; Columbia University; Johns Hopkins University; the University of Minnesota; Northwestern University, and the University of California – Los Angeles). All underwent anthropomorphic measurement and extensive evaluation by questionnaires at baseline, followed by 4 subsequent examinations at intervals of approximately 2-4 years. Age and sex were self-reported.

ECGs were recorded in the supine position after a period of rest. MESA ECG data were collected using GE MAC 1200 electrocardiographs. Digitally collected ECGs were transferred via phone lines to the MESA ECG center (EPICARE). The ECGs were automatically processed by use of GE Marquette 12-SL software (2001 version), after visual inspection of the recordings for quality.

Current AF at baseline was an exclusion criterion. Follow-up phone calls to study participants (every 9-12 months) were used to identify all hospitalizations. Medical records, including discharge diagnoses, were obtained for each hospitalization. Incident AF was defined by International Classification of Disease codes 427.31 or 427.32 (9th revision). In addition, new diagnoses of AF were identified at follow-up by the presence of AF or atrial flutter on a study ECG at Exam 5 (approximately 10 years after baseline).

Further information can be found at:

<http://www.ncbi.nlm.nih.gov/projects/gap/cgi-bin/study.cgi?study_id=phs000209.v13.p3>

**PREVEND**

The PREVEND cohort study was founded in 1997, and is an ongoing community-based cohort study including 8592 inhabitants of the city of Groningen, The Netherlands.[^6^](#_ENREF_6) PREVEND is investigating the natural course of microalbuminaria and its relation to renal and cardiovascular disease. Details of the protocol, AF ascertainment and covariate definitions have been described elsewhere ([www.prevend.org](http://www.prevend.org/)). AF was ascertained if either atrial flutter or AF was present on a 12-lead ECG obtained at one of the three PREVEND follow-up visits, or at an outpatient visit or hospital admission in the two hospitals in the city of Groningen (University Medical Center Groningen and Martini Hospital). Systolic and diastolic blood pressures were calculated as the mean of the last two measurements of the two visits, using an automatic GE Dinamap XL Model 9300 series device. Hypertension was defined as systolic blood pressure >140 mmHg, diastolic blood pressure >90 mmHg, or use of antihypertensive drugs. Use of antihypertensive drugs was based on available information from the pharmacy prescription database. Body mass index (BMI) was calculated as the ratio of weight to height squared (kg/m^2^). Genotyping was performed using the Illumina CytoSNP12v2 array. Genotype calling was performed using GenomeStudio, and imputation was performed using Beagle with the HapMap release 22 CEU referent panel. Analyses were performed with Plink v1.07 and R.

***Replication studies***

**BEAT-AF/GAPP**

The Basel Atrial Fibrillation Cohort Study (BEAT-AF) is a prospective observational, multicenter cohort study. Between 2010 and 2014, 1550 patients with documented AF were enrolled across 7 centers in Switzerland. Exclusion criteria were the inability to sign informed consent and the presence of short transient forms of AF. At baseline, patients completed detailed questionnaires about personal, medical, nutritional and lifestyle factors, current AF symptoms and co-morbidities. Current medications were recorded. A resting 12-lead electrocardiogram (ECG) was recorded and all patients underwent venous blood sampling at the local study center, including DNA from leukocytes. Yearly follow-ups by mailed questionnaires and phone interviews were performed in all patients in order to collect similar information as at baseline and to obtain details about adverse events.

Controls were enrolled from the ‘genetic and phenotypic determinants of blood pressure and other cardiovascular risk factors’ (GAPP) study, which is an ongoing prospective population-based cohort study among healthy adults in the Principality of Liechtenstein. Between 2010 and 2013, all inhabitants of the Principality of Liechtenstein aged between 25 and 41 years were invited and 2170 agreed to participate in the study. Main exclusion criteria were established cardiovascular disease, chronic kidney disease, diagnosed sleep apnea, a body mass index (BMI) > 35 kg/m^2^, intake of antidiabetic drugs or any other severe illness. Examinations included detailed assessment of personal, medical, lifestyle and nutritional factors, standardized assessment of weight, height and waist circumference, blood pressure measurement, electrocardiography, bioimpedance analysis, blood, urinary and genetic sampling, spirometry and sleep pulse oximetry with nasal flow measurement. Follow-up examinations are scheduled every 3-5 years. The detailed study design has previously been published ( <http://www.ncbi.nlm.nih.gov/pubmed/23299990> )

**FINCAVAS**

The purpose of the Finnish Cardiovascular Study (FINCAVAS) is to construct a risk profile - using genetic, haemodynamic and electrocardiographic (ECG) markers - of individuals at high risk of cardiovascular diseases, events and deaths. All patients scheduled for an exercise stress test at Tampere University Hospital, who gave informed consent to participate, were recruited between October 2001 and December 2007. The total number of participants was 4,567. In addition to repeated measurements of heart rate and blood pressure, digital high-resolution ECG at 500 Hz was recorded continuously during the entire exercise test, including the resting and recovery phases. About 20% of the patients were examined with coronary angiography. Genetic variations known or suspected to alter cardiovascular function or pathophysiology were analyzed to elucidate the effects and interactions of these candidate genes, exercise, and commonly used cardiovascular medications.

**Malmo Diet and Cancer Study (MDCS)**

MDCS is a community-based prospective epidemiologic cohort of middle-aged individuals from Southern Sweden.[^7^](#_ENREF_7) In total, 30,447 subjects attended a baseline exam in 1991-1996, when they filled out a questionnaire and underwent anthropometric and blood pressure measurements. Hypertension was defined as self-reported use of antihypertensive medications or measured blood pressure ≥140/90 mmHg. Prevalent or incident cases of atrial fibrillation (AF), heart failure and ischemic heart disease were ascertained from nation-wide hospital registers with high validity as described previously.[^8^](#_ENREF_8) Genome-wide genotyping of single nucleotide variants was performed using the Illumina Human Omni Express Exome BeadChip kit. Genotyping was performed in a nested case-cohort design, including a random subset of 7255 subjects with complete data and 876 cases with incident AF. Imputation was performed to genotypes in the 1000 Genomes Project phase 1 using IMPUTE.

**UK Biobank**

Details of genotyping, imputation, and calculation of principal components of ancestry in the UK biobank interim dataset can be found on the UK biobank website (<http://www.ukbiobank.ac.uk/>). Briefly, samples were genotyped either by UK BiLEVE Axiom array (UKBL) or UK Biobank Axiom array (UKBB). Both arrays include ~800,000 SNPs and more than 95% of common marker contents are similar. Imputation was phased by modified version of SHAPEIT2 and imputed by IMPUTE2, using a combined panel of UK10K haplotype and 1000G phase 3 as the reference panel. All significant variants detected in the discovery study passed quality control filters in the UK biobank data (imputation quality info ≥ 0.4, variant missing rate < 5%, individual missing rate < 10%, and variant genotype probability > 0.9 in > 90% of the individuals). Variants were then transformed to hard-called genotypes (probability threshold ≥ 0.9, minor allele frequency (MAF) ≥ 0.01, and missing rate per variant <5%). We used logistic regression to test the association between each hard-called variant and risk of AF using an additive genetic model, adjusting for baseline age, sex, array, and the first 5 principal components of ancestry. Quality control, transformation and analyses were performed by QCTOOL and Plink v1.90b. This research has been conducted using the UK Biobank Resource under Application Number 17488.

**Supplemental Table 1**. Analysis-specific numbers of included autosomal SNPs and genomic inflation estimates by study sample.

Abbreviations: λ, genomic inflation factor.

|  | Age | | | | Sex | | Body mass index | | Hypertension | |
| --- | --- | --- | --- | --- | --- | --- | --- | --- | --- | --- |
|  | ≤65 years | | >65 years | |  | |  |  |  | |
| Cohort | N SNPs | (λ) | N SNPs | (λ) | N SNPs | (λ) | N SNPs | (λ) | N SNPs | (λ) |
| ***Incident*** |  |  |  |  |  |  |  |  |  |  |
| AGES | — | — | — | — | 2136078 | 0.995 | 2136115 | 1.060 | 2101296 | 1.033 |
| ARIC | 2165687 | 0.987 | — | — | 2179076 | 1.004 | 2179099 | 1.169 | 2179081 | 1.012 |
| CHS | — | — | 2067494 | 1.045 | 2067674 | 1.027 | 2067674 | 1.121 | 2067674 | 1.021 |
| FHS | — | — | 2144443 | 1.004 | 2144443 | 1.005 | 2144443 | 1.061 | 2144439 | 0.994 |
| MESA | — | — | 2093768 | 1.024 | 2171564 | 1.039 | 2171941 | 1.061 | 2171884 | 1.033 |
| PREVEND | — | — | — | — | 1859130 | 1.040 | 1859123 | 1.105 | 1858235 | 1.006 |
| PROSPER | — | — | 2163841 | 1.014 | 2163841 | 0.998 | 2163841 | 1.004 | 2163841 | 1.006 |
| RS | 2164948 | 1.022 | 2165443 | 1.20 | 2165273 | 1.002 | 2165437 | 0.992 | 2165217 | 1.008 |
| WGHS | 2159805 | 1.023 | 2159805 | 1.001 | — | — | 2159805 | 1.032 | 2159805 | 0.995 |
| ***Prevalent*** |  |  |  |  |  |  |  |  |  |  |
| AFNET/KORA | 2168049 | 1.017 | — | — | 2168048 | 0.988 | 2168003 | 1.028 | 2168050 | 1.030 |
| AGES | — | — | — | — | 2133548 | 0.998 | 2126844 | 1.033 | 2133550 | 1.060 |
| BioVU 660 | — | — | — | — | 1658797 | 1.015 | 1660844 | 0.846 | 1625789 | 0.951 |
| BioVU o1 | — | — | 1813638 | 1.021 | 1813587 | 1.008 | 1813746 | 0.991 | 1803269 | 0.984 |
| CCAF | 2176365 | 1.036 | 2173984 | 1.027 | 2175987 | 1.001 | 2175951 | 1.014 | 2175987 | 1.024 |
| FHS | — | — | 2144443 | 1.031 | 2144393 | 1.009 | 2143524 | 1.009 | 2144443 | 1.031 |
| LURIC | 2169011 | 1.005 | 2169011 | 1.003 | 2169007 | 0.999 | 2169003 | 1.014 | 2163105 | 1.022 |
| MGH/MIGEN | 2166720 | 1.004 | — | — | 2167717 | 0.967 | — | — | — | — |
| RS | — | — | 2164958 | 1.021 | 2165408 | 1.011 | 2165435 | 1.019 | 2165315 | 1.000 |
| SHIP | — | — | — | — | 2237384 | 1.036 | 2239095 | 1.075 | 2238479 | 1.033 |

**Supplemental Table 2.** Sixteen AF-associated loci reported in previous association studies.

| **Locus** | **SNP** | **Closest gene** | **Minor allele**  **frequency (%)** | **Relative risk**  **(95% CI)** | ***P* Value** | **Reference** |
| --- | --- | --- | --- | --- | --- | --- |
| 1q21 | rs6666258 | *KCNN3* | 29.9 | 1.18 (1.13-1.23) | 2.0x10^-14^ | [^1^](#_ENREF_1) |
| 1q24 | rs3903239 | *PRRX1* | 44.7 | 1.14 (1.10-1.18) | 9.1x10^-11^ | [^1^](#_ENREF_1) |
| 3p25 | rs4642101 | *CAND2* | 35 | 1.10 (1.06-1.14) | 9.8x10^-9^ | [^2^](#_ENREF_2) |
| 4q25 | rs1448818 | *PITX2* | 25 | 1.12 (1.08-1.17) | 1.6x10^-8^ | [^9^](#_ENREF_9) |
| 4q25 | rs6817105 | *PITX2* | 13.1 | 1.64 (1.55-1.73) | 1.8x10^-74^ | [^1^](#_ENREF_1) |
| 4q25 | rs4400058 | *PITX2* | 9 | 1.28 (1.21-1.36) | 2.2x10^-16^ | [^9^](#_ENREF_9) |
| 4q25 | rs6838973 | *PITX2* | 44 | 1.11 (1.08-1.15) | 6.0x10^-9^ | [^9^](#_ENREF_9) |
| 6q22 | rs13216675 | *GJA1* | 31 | 1.10 (1.06-1.14) | 2.2x10^-8^ | [^2^](#_ENREF_2) |
| 7q31 | rs3807989 | *CAV1* | 40.4 | 0.88 (0.84-0.91) | 9.6x10^-11^ | [^1^](#_ENREF_1) |
| 9q22 | rs10821415 | *C9orf3* | 42.4 | 1.13 (1.08-1.18) | 7.9x10^-9^ | [^1^](#_ENREF_1) |
| 10q22 | rs10824026 | *SYNPO2L* | 15.8 | 0.85 (0.81-0.90) | 1.7x10^-8^ | [^1^](#_ENREF_1) |
| 10q24 | rs12415501 | *NEURL* | 16 | 1.18 (1.13-1.23) | 6.5x10^-16^ | [^2^](#_ENREF_2) |
| 12q24 | rs10507248 | *TBX5* | 27 | 1.12 (1.08-1.16) | 5.7x10^-11^ | [^2^](#_ENREF_2) |
| 14q23 | rs1152591 | *SYNE2* | 47.6 | 1.13 (1.09-1.18) | 6.2x10^-10^ | [^1^](#_ENREF_1) |
| 15q24 | rs7164883 | *HCN4* | 16.0 | 1.16 (1.10-1.22) | 1.3x10^-8^ | [^1^](#_ENREF_1) |
| 16q22 | rs2106261 | *ZFHX3* | 17.6 | 1.24 (1.17-1.30) | 3.2x10^-16^ | [^1^](#_ENREF_1) |

**Supplemental Table 3.** Details of study populations.

| ***Discovery Cohorts*** | |  |  |  |  |
| --- | --- | --- | --- | --- | --- |
| **Cohort** | **Country** | **Study design** | **AF diagnosis** | **Hypertension Definition** | **Reference** |
| **AFNET/KORA** | Germany | Case-Control | ECG | >140mmHg, or diastolic blood pressure >90mmHg, or use of antihypertensive medication for this purpose |  |
| **AGES** | Iceland | Cohort | ICD-10: I48 | Hypertension, either self reported from qustionnaire, derived from physiological measurements (systolic blood pressure, diastolic blood pressure ), or use of hypertension medications | [^12^](#_ENREF_12) |
| **ARIC** | United States | Cohort | ECG at study visits, hospital discharge codes or death certificates (ICD-9 code 427.31 or 427.32, or ICD-10 code I48). | SBP>=140, DBP>=90 or use of antihypertensive meds |  |
| **BioVU** | United States | Cohort: hospital-based study (Biobank) | >18 years and an ICD-9 diagnosis for AF or flutter (ICD-9: 427.3, 427.31, and 427.32), or a cardiologist diagnosis of AF as identified by a natural language processing tool from the unstructured free text of the ECG impression. In all instances, patients with a history of a heart transplant were excluded (Current Procedural Terminology: 33935, 3394, and 580; ICD-9: V42.1, 996.83) | Hypertension was identified from the EHR using a multimodal algorithm using blood pressures, billing codes, medications, and text mentions of hypertension. |  |
| **CHS** | United States | Cohort | Annual CHS ECG or by ICD-9 code on a hospital discharge. | SBP>=140, DBP>=90 or use of antihypertensive meds for treatment of high blood pressure | [^16^](#_ENREF_16) |
| **CCFS** | United States | Case-control | ECG or medical report | SBP>=140, DBP>=90 or history of hypertension | [^1^](#_ENREF_1) |
| **FHS** | United States | Cohort | Cardiovascular hospital and outside records and ECG at all FHS examinations | SBP>=140, DBP>=90 or use of antihypertensive meds |  |
| **LURIC** | Germany | Cohort: hospital-based study | ECG or medical report | SBP>=140, DBP>=90 or history of hypertension | [^19^](#_ENREF_19) |
| **MESA** | United States | Cohort | All interim hospital admissions, cardiovascular outpatient diagnoses, and deaths. More information is included in the cohort description. | Self-reported treatment for hypertension or a systolic BP ≥140 mm Hg or diastolic BP ≥90 mm Hg |  |
| **MGH/MIGEN** | United States | Cohort: hospital-based study | ECG | - |  |
| **PREVEND** | The Netherlands | Cohort | ECG | Hypertension was defined as systolic blood pressure >140 mm Hg, diastolic blood pressure >90 mm Hg, or use of antihypertensive drugs |  |
| **PROSPER** | Ireland, Scotland, and the Netherlands | Randomized, double-blind, placebo-controlled trial | ECG | systolic blood pressure was ≥ 140 mm Hg or the diastolic blood pressure was ≥ 90 mm Hg | [^25^](#_ENREF_25) |
| **Rotterdam Study** | The Netherlands | Cohort | Study visit ECG, hospital discharge information, and general practitioner diagnoses. | SBP ≥ 140 mmHg and/or DBP ≥ 90 mmHg or antihypertensive medication |  |
| **SHIP** | Germany | Cohort | Study visit ECG or tele-ecg | SBP ≥ 140 mmHg and/or DBP ≥ 140 mmHg or antihypertensive medication | [^28^](#_ENREF_28) |
| **WGHS** | United States | Cohort | ECG or medical report | History of hypertension ≥ 140/90, defined as MD diagnosis at enrollment or run-in OR SBP ≥ 140 OR DBP ≥ 90 | [^29-31^](#_ENREF_29) |
| ***Replication Cohorts*** | |  |  |  | |
| **Cohort** | **Country** | **Study design** | **AF diagnosis** | **Hypertension Definition** | **Reference** |
| **Beat-AF/ GAPP** | Switzerland / Liechtenstein | Case-control | ECG | ≥140/90 mmHg or use of BP lowering drugs | [^32^](#_ENREF_32) |
| **FINCAVAS** | Finland | Cohort: hospital-based study | ECG, hospital discharge information | ≥140/90 mmHg or use of BP lowering drugs | [^33^](#_ENREF_33) |
| **MDCS** | Sweden | Case-control | National register | ≥140/90 | [^7^](#_ENREF_7) |
| **UK Biobank** | United Kingdom | Cohort: Biobank | Verbal interview, diagnosis codes, or procedure codes | On anti-hypertensive medication at enrollment |  |

**Supplemental Table 4.** Details regarding study genotyping and data analysis.

| ***Discovery Cohorts*** | | | | | | | |
| --- | --- | --- | --- | --- | --- | --- | --- |
| **Study** | **Genotyping platform** | **Imputation software** | **Imputation Backbone / NCBI Build** | **Statistical software** | **GWAS Statistical Analysis** | **Covariates** | **Robust variance estimator**  **(Y/N)** |
| **AFNET/KORA** | Published data[^9^](#_ENREF_9) | | | ProbABEL, R | Logistic regression | Age, sex, 4 PCs | Y |
| **AGES** | Published data[^9^](#_ENREF_9) | | | ProbABEL, R | Inc: Cox proportional hazard regression; Pre: Logistic regression | Age, sex | Y |
| **ARIC** | Published data[^9^](#_ENREF_9) | | | ProbABEL, R | Cox proportional hazard regression | Age, sex, center | N |
| **BioVU** | Human660W-Quad BeadChip / HumanOmni1-Quad BeadChip | SHAPE-IT/IMPUTE2 | 1000G Phase 1 integrated v3 panel  /b37 | PLINK | Logistic regression | Age, sex, 2PCs | N |
| **CHS** | Published data[^9^](#_ENREF_9) | | | R | Cox proportional hazard regression | Age, sex, clinic | N |
| **CCFS** | Published data[^9^](#_ENREF_9) | | | ProbABEL, R | Logistic regression | Age, sex, 3 PCs + CAD | Y |
| **FHS** | Published data[^9^](#_ENREF_9) | | | R | Inc: Cox proportional hazard regression; Pre: GEE | Age, sex | Y |
| **LURIC** | Published data[^9^](#_ENREF_9) | | | ProbABEL | Logistic regression | Age, sex, 3 PCs | Y |
| **MESA** | Affymetrix Genome-Wide Human SNP Array 6.0 (Affymetrix, Santa Clara, CA) | IMPUTE2 | 1,000 Genomes Phase I integrated variant set (NCBI build 37 / hg19) | ProbABEL, R | Logistic regression | Age, sex, sites, 2PCs | Y |
| **MGH/MIGEN** | Published data[^9^](#_ENREF_9) | | | R | Logistic regression | Age, sex, 6 PCs | N(age), Y(sex) |
| **PREVEND** | Illumina CytoNP12 v2 | SHAPE-IT/IMPUTE2 | 1000G Phase 1 integrated v3 panel/b37 | ProbABEL, R | Logistic regression | Age,sex, PC1-5 | N |
| **PROSPER** | Published data[^9^](#_ENREF_9) | | | ProbABEL, R | Inc: Cox proportional hazard regression | Age, sex, PC 1-4 | N |
| **Rotterdam Study** | Published data[^9^](#_ENREF_9) | | | ProbABEL, R | Inc: Cox proportional hazard regression; Pre: Logistic regression | Age, sex | N |
| **SHIP** | Published data[^9^](#_ENREF_9) | | | ProbABEL | Logistic regression | age, sex | Y |
| **WGHS** | Published data[^9^](#_ENREF_9) | | | ProbABEL | Cox PH regression (incident AF) | age, eigenvector E2 for HXHTN interaction only | N(age), Y(sex, bmi) |
| ***Replication Cohorts*** | | | | | | | |
| **Study** | **Genotyping platform** | **Imputation software** | **Imputation Backbone / NCBI Build** | **Statistical software** | **GWAS Statistical Analysis** | **Covariates** | **Robust variance estimator**  **(Y/N)** |
| **Beat-AF/GAPP** | Illumina HumanCoreExome | SHAPEIT v2.r790 + IMPUTE v.2.3.2 | Build 37 | R | Logistic regression | Sex, 10 PCs | N |
| **FINCAVAS** | Illumina Metabochip and Human CoreExome | SHAPEIT v2, IMPUTE v2.3.0 | 1000G Phase 1 integrated v3 panel  /b37 | R | Logistic regression | Age, sex and 10 PCs | N |
| **MDCS** | Illumina Human Omni Express Exome BeadChip | IMPUTE | NCBI Build 37 | R | Cox proportional hazard regression | Age, sex and 10 PCs | N |
| **UK Biobank** | UK Bileve or UK Biobank Axiom Array | IMPUTE | Combined 1000G and UK10K / Hg37 | R | Logistic regression | Age, sex, and first 5 PCs | N |

**Supplemental Figure 1a-1d.** Quantile-quantile plots for each genetic interaction analysis on chromosomes 1-22.


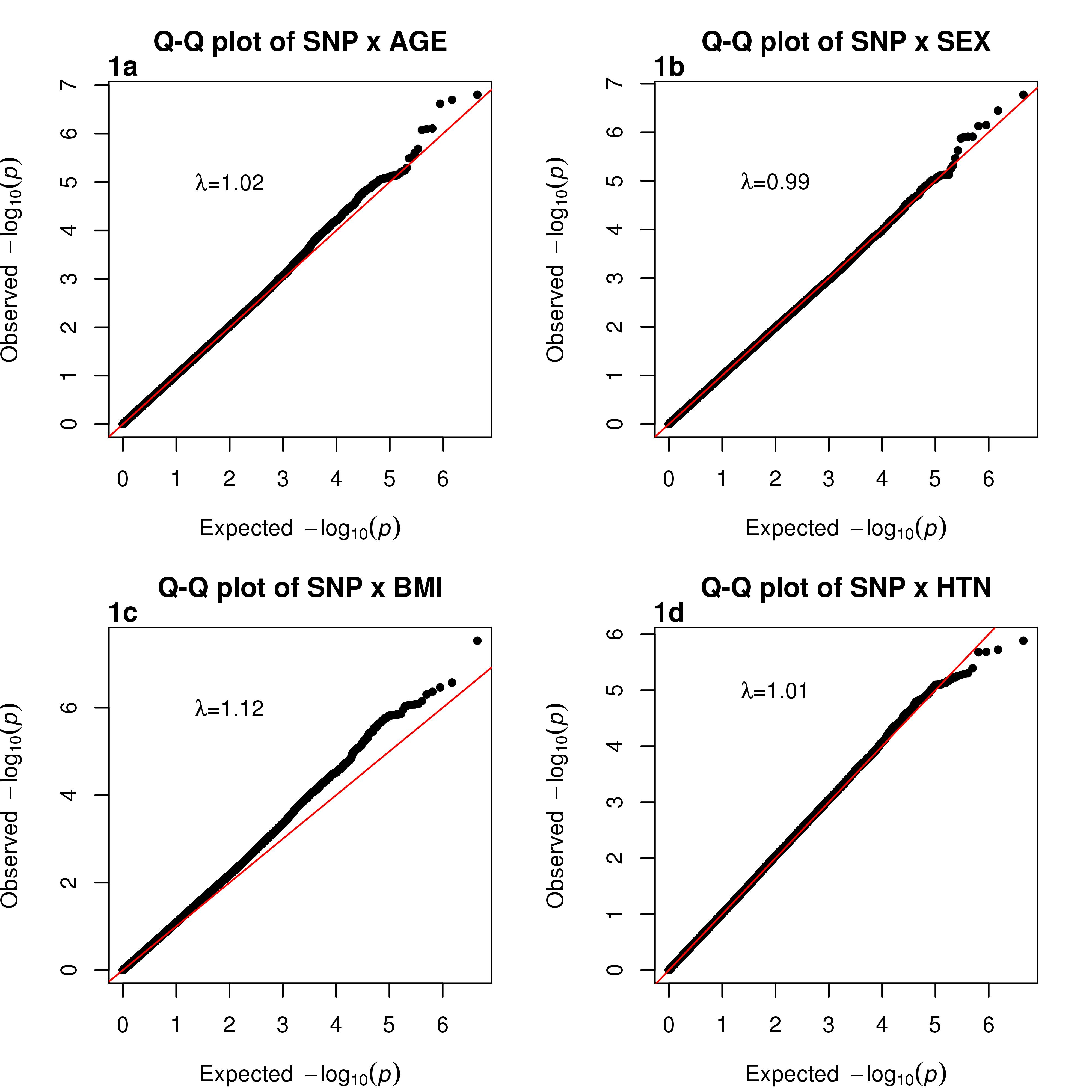


**Supplemental Figure 2.** Regional association plot for genome-wide significant SNP by BMI interaction on chromosome 10.

**Supplemental** **References**

1 Ellinor, P. T. *et al.* Meta-analysis identifies six new susceptibility loci for atrial fibrillation. *Nature genetics* **44**, 670-675 (2012).

2 Sinner, M. F. *et al.* Integrating genetic, transcriptional, and functional analyses to identify five novel genes for atrial fibrillation. *Circulation*, CIRCULATIONAHA-114 (2014).

3 Roden, D. M. *et al.* Development of a large‐scale de‐identified DNA biobank to enable personalized medicine. *Clinical Pharmacology & Therapeutics* **84**, 362-369 (2008).

4 Pulley, J., Clayton, E., Bernard, G. R., Roden, D. M. & Masys, D. R. Principles of human subjects protections applied in an opt‐out, de‐identified biobank. *Clinical and translational science* **3**, 42-48 (2010).

5 Weeke, P. *et al.* Examining Rare and Low-Frequency Genetic Variants Previously Associated With Lone or Familial Forms of Atrial Fibrillation in an Electronic Medical Record System A Cautionary Note. *Circulation: Cardiovascular Genetics* **8**, 58-63 (2015).

6 Vermond, R. A. *et al.* Incidence of atrial fibrillation and relationship with cardiovascular events, heart failure, and mortality: a community-based study from the Netherlands. *Journal of the American College of Cardiology* **66**, 1000-1007 (2015).

7 Smith, J. G., Platonov, P. G., Hedblad, B., Engstrom, G. & Melander, O. Atrial fibrillation in the Malmo Diet and Cancer study: a study of occurrence, risk factors and diagnostic validity. *European journal of epidemiology* **25**, 95-102, doi:10.1007/s10654-009-9404-1 (2010).

8 Smith, J. G. *et al.* Assessment of conventional cardiovascular risk factors and multiple biomarkers for the prediction of incident heart failure and atrial fibrillation. *Journal of the American College of Cardiology* **56**, 1712-1719 (2010).

9 Lubitz, S. A. *et al.* Novel genetic markers associate with atrial fibrillation risk in Europeans and Japanese. *Journal of the American College of Cardiology* **63**, 1200-1210 (2014).

10 Nabauer, M. *et al.* The Registry of the German Competence NETwork on Atrial Fibrillation: patient characteristics and initial management. *Europace : European pacing, arrhythmias, and cardiac electrophysiology : journal of the working groups on cardiac pacing, arrhythmias, and cardiac cellular electrophysiology of the European Society of Cardiology* **11**, 423-434 (2009).

11 Wichmann, H. E., Gieger, C., Illig, T. & group, M. K. s. KORA-gen-resource for population genetics, controls and a broad spectrum of disease phenotypes. *Das Gesundheitswesen* **67**, 26-30 (2005).

12 Harris, T. B. *et al.* Age, Gene/Environment Susceptibility–Reykjavik Study: multidisciplinary applied phenomics. *American journal of epidemiology* **165**, 1076-1087 (2007).

13 The Atherosclerosis Risk in Communities (ARIC) Study: design and objectives. The ARIC investigators. *Am J Epidemiol* **129**, 687-702 (1989).

14 Alonso, A. *et al.* Incidence of atrial fibrillation in whites and African-Americans: the Atherosclerosis Risk in Communities (ARIC) study. *American heart journal* **158**, 111-117 (2009).

15 Teixeira, P. L. *et al.* Evaluating electronic health record data sources and algorithmic approaches to identify hypertensive individuals. *Journal of the American Medical Informatics Association*, ocw071 (2016).

16 Fried, L. P. *et al.* The cardiovascular health study: design and rationale. *Annals of epidemiology* **1**, 263-276 (1991).

17 Dawber, T. R., Meadors, G. F. & Moore Jr, F. E. Epidemiological Approaches to Heart Disease: The Framingham Study*. *American Journal of Public Health and the Nations Health* **41**, 279-286 (1951).

18 Kannel, W. B., Feinleib, M., McNamara, P. M., Garrison, R. J. & Castelli, W. P. An investigation of coronary heart disease in families The Framingham offspring study. *American journal of epidemiology* **110**, 281-290 (1979).

19 Winkelmann, B. R. *et al.* Rationale and design of the LURIC study-a resource for functional genomics, pharmacogenomics and long-term prognosis of cardiovascular disease. *Pharmacogenomics* **2**, S1-S73 (2001).

20 Ambale-Venkatesh, B. *et al.* Diastolic function assessed from tagged MRI predicts heart failure and atrial fibrillation over an 8-year follow-up period: the multi-ethnic study of atherosclerosis. *European Heart Journal-Cardiovascular Imaging* **15**, 442-449 (2014).

21 Bild, D. E. *et al.* Multi-ethnic study of atherosclerosis: objectives and design. *American journal of epidemiology* **156**, 871-881 (2002).

22 Ellinor, P. T., Yoerger, D. M., Ruskin, J. N. & MacRae, C. A. Familial aggregation in lone atrial fibrillation. *Human genetics* **118**, 179-184 (2005).

23 Kathiresan, S. *et al.* Genome-wide association of early-onset myocardial infarction with single nucleotide polymorphisms and copy number variants. *Nature genetics* **41**, 334-341 (2009).

24 Heerspink, H. J. L. *et al.* Albuminuria assessed from first-morning-void urine samples versus 24-hour urine collections as a predictor of cardiovascular morbidity and mortality. *American journal of epidemiology* **168**, 897-905 (2008).

25 Shepherd, J. *et al.* The design of a prospective study of pravastatin in the elderly at risk (PROSPER). *The American journal of cardiology* **84**, 1192-1197 (1999).

26 Heeringa, J. *et al.* Prevalence, incidence and lifetime risk of atrial fibrillation: the Rotterdam study. *European heart journal* **27**, 949-953 (2006).

27 Hofman, A. *et al.* The Rotterdam Study: objectives and design update. *European journal of epidemiology* **22**, 819-829 (2007).

28 John, U. *et al.* Study of Health In Pomerania (SHIP): a health examination survey in an east German region: objectives and design. *Sozial-und Präventivmedizin* **46**, 186-194 (2001).

29 Conen, D. *et al.* Alcohol consumption and risk of incident atrial fibrillation in women. *Jama* **300**, 2489-2496 (2008).

30 Rexrode, K. M., Lee, I. M., Cook, N. R., Hennekens, C. H. & Buring, J. E. Baseline characteristics of participants in the Women's Health Study. *Journal of women's health & gender-based medicine* **9**, 19-27 (2000).

31 Ridker, P. M. *et al.* Rationale, design, and methodology of the Women’s Genome Health Study: a genome-wide association study of more than 25 000 initially healthy American women. *Clinical chemistry* **54**, 249-255 (2008).

32 Christophersen, I. E. *et al.* Familial aggregation of atrial fibrillation a study in Danish twins. *Circulation: Arrhythmia and Electrophysiology* **2**, 378-383 (2009).

33 Nieminen, T. *et al.* The Finnish Cardiovascular Study (FINCAVAS): characterising patients with high risk of cardiovascular morbidity and mortality. *BMC cardiovascular disorders* **6**, 9 (2006).
